# Effects of a dolphin interaction program on children with autism spectrum disorders – an exploratory research

**DOI:** 10.1186/1756-0500-5-199

**Published:** 2012-04-26

**Authors:** Emílio Salgueiro, Laura Nunes, Alexandra Barros, João Maroco, Ana Isabel Salgueiro, Manuel E dos Santos

**Affiliations:** 1 Unidade de Investigação em Psicologia e Saúde. ISPA – Instituto Universitário, Rua Jardim do Tabaco, 44, 1149-041, Lisbon, Portugal; 2 Hospital de Faro, Faro, Portugal; 3 Hospital de Santa Maria, Lisbon, Portugal; 4 Apoiar – Consultadoria em Saúde Mental e Linguagem, Lda., Lisbon, Portugal; 5 Unidade de Investigação em Eco-etologia. ISPA – Instituto Universitário, Rua Jardim do Tabaco, 44, 1149-041, Lisbon, Portugal

## Abstract

**Background:**

Interaction programs involving dolphins and patients with various pathologies or developmental disorders (e.g., cerebral palsy, intellectual impairment, autism, atopic dermatitis, post-traumatic stress disorder, depression) have stimulated interest in their beneficial effects and therapeutic potential. However, the true effects observed in different clinical and psycho-educational setups are still controversial.

**Results:**

An evaluation protocol consisting of the Childhood Autism Rating Scale (CARS), Psychoeducational Profile-Revised (PEP-R), Autism Treatment Evaluation Checklist (ATEC), Theory of Mind Tasks (ToM Tasks) and a custom-made Interaction Evaluation Grid (IEG) to evaluate behavioural complexity during in-pool interactions was applied to 10 children diagnosed with Autism Spectrum Disorders. The ATEC, ToM Tasks and CARS results show no benefits of the dolphin interaction program. Interestingly, the PEP-R suggests some statistically significant effects on ‘Overall development score’, as well as on their ‘Fine motor development’, ‘Cognitive performance’ and ‘Cognitive verbal development’. Also, a significant evolution in behavioural complexity was shown by the IEG.

**Conclusions:**

This study does not support significant developmental progress resulting from the dolphin interaction program.

## Background

Interaction programs involving dolphins and patients with various pathologies or developmental disorders (e.g., cerebral palsy, intellectual impairment, autism, atopic dermatitis, post-traumatic stress disorder, depression) have stimulated interest in their beneficial effects and therapeutic potential e.g.,[1-8].

The choice of dolphins for this interaction program with ASD children and many other such programs has been based on a number of factors namely: positive image of these animals in the general population (big, protective, friendly aquatic mammals, intelligent and communicative); curious, easily and willingly trainable; capable of sustaining complex interaction with humans when properly conditioned; general cooperative and playful attitude; accepting physical contact, including hugs, caresses and kisses; non-threatening expression; soft skin, and delicate movements. These factors have been suggested as useful, in facilitating the establishment of their relationships with humans, with possible therapeutic effects in children [9,10].

In spite of the popular appeal, scientific evaluation of this therapeutic strategy has been scarce and controversial. “Dolphin Assisted Therapy” (DAT) has been examined in a relatively low number of studies, almost always affected by severe methodological limitations [11-15]. Even though stringent methodological criteria should be the norm in science (see, for example, Marino & Lilienfeld [11]), it is impossible to observe the effect of DAT-alone in clinical scenarios (i.e., treating ASD children only with dolphins, ceasing all other therapies and developmental programs they may be following). Moreover, some results of (even suboptimal) interaction programs are clinically interesting and deserve further attention [16].

Children with Autism spectrum disorders (ASD) have reduced capacity for social interactions, such as mutual gaze, pointing, showing objects of interest and answering back when called [17]. They show a lack of emotional resonance that disturbs the translation and interpretation of the emotions of others. These limitations are linked to their difficulties in the establishment of primary and secondary intersubjectivities, conducive to a richer communication and to the development of language [18-22].

The goal of this study was to explore the possibility that developmental progress in ASD children (namely, in communication and social skills) might be improved by an in-water complex interaction program with dolphins and humans. This research-oriented, non-commercial interaction program was created and implemented at a dolphinarium, with the cooperation of the local education authority in referring the ASD children.

## Methods

### Participants

Ten children, registered in December 2002 as ASD at the Regional Education Authority of the Algarve (Southern Portugal) and whose families agreed to participate in the full program, which was carried out at the marine park Zoomarine, near Albufeira, Portugal. The age of these children at the time of their first evaluation ranged from 3 years and 6 months to 13 years and 6 months. Mean age was 6 years and 9 months (SD = 2 years and 9 months). There were eight boys and two girls, one girl being African and the remaining nine children Caucasian. One child presented an Autism Disorder and the other nine Pervasive Developmental Disorders Not Otherwise Specified [23]. They all lived at home with their families, and all completed the program. Written consent was obtained from all parents of participating children.

### Instruments

Subjects were evaluated using the following instruments:

1. *CARS.* This scale (*Childhood Autism Rating Scale*, [24]) is composed of 15 items, which were grouped in 5 factors following Stella et al. (1999): *Emotional Reactivity* (Activity level, Body use, Emotional response, Object use, Fear or nervousness, General impressions), *Social Communication* (Imitation, Adaptation to change, Nonverbal communication, Verbal communication, Relating to people), *Social Orienting* (Listening response, Visual response, Relating to people), *Odd Sensory Exploration* (Taste, Touch and Smell use) and *Cognitive and Behavioural Consistency* (Consistency of intellectual response, General impressions, Fear or nervousness). CARS data analysis was performed both on the item scores and on the factor scores obtained by their summation [25].

2. *PEP-R*. This psychoeducational developmental test for ASD children (*Psychoeducational Profile Revised*, [26]), has 11 sub-scales: seven *Developmental* subscales (Imitation, Perception, Fine and Gross motor coordination, Eye-hand integration, Cognitive performance and Cognitive verbal skills, plus an Overall development score) and four *Behavioural* sub-scales (Cooperation and human interest, Play and interest in materials, Sensory responses and Language).

3. *ATEC*. This tool (*Autism Treatment Evaluation Checklist*, [27]) was specifically designed for the evaluation of the effects of therapies on children with ASD. Its four sub-scales are Speech, Sociability, Sensory/Cognitive awareness and Health/Physical/Behaviour, plus an Overall development score).

4. *A custom-designed developmental protocol to evaluate the child’s capacity for a ‘Theory of Mind’ (ToM)*. The material developed by Hadwin, Baron-Cohen, Howlin & Hill [28] and Howlin, Baron-Cohen & Hadwin [29] to teach children to “mind-read” was adapted in the present study as an evaluation tool. It consisted of a five-level procedure of increasing difficulty in the recognition tasks: I - recognizing facial expressions from photographs; II - recognizing emotions from schematic drawings; III- identifying “situation-based” emotions; IV - identifying “desire-based” emotions; V - identifying “belief-based” emotions. Since the intention was to evaluate the children’s current ability to recognize emotional states (and not to teach this ability), the protocol was reduced from the original 144 pictures to 38. Four pictures were maintained in levels I and II. For levels III and IV, random selections of 12 and 6 pictures, respectively, were made. In level V, 12 pictures were chosen, each requiring two answers. Results were recorded in a weighted success/failure table, with final scores that could range from 0 to 252 (Additional file 1: Appendix A).

5. *A custom-designed ‘Interaction Evaluation Grid’*. This grid is composed of 51 behavioural items, generally of increasing complexity, used to rate the children’s behaviour during each interaction session (Additional file 2: Appendix B).

### Procedures

Ethical approval for this research project was obtained from research director of ISPA-Instituto Universitário, the Marine park’s administration and curators and the Regional Education Board of the Algarve.

During the research period (February 2003 – February 2005), the ten children underwent 6 evaluation sessions, three before the interaction program (see below), and three after the program, with intervals between each successive follow-up of three or four months, to evaluate the persistence of any behavioural change.

**Figure 1:**
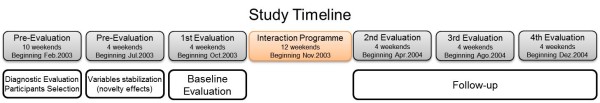
Study Timeline.

The total interaction and evaluation program took place during 42 non-consecutive weekends (due to park impediments). In the first 10 weekends, participants were selected and diagnostic evaluations were made (see Figure 1). In order to stabilize variables and to reduce the novelty effect, children were pre-evaluated (4 weekends) and, right before the interaction program, a proper evaluation allowed us to collect the baseline data (4 weekends). The dolphin interactions occurred for 12 weekly sessions and subsequently, 3 evaluations were carried out to obtain the follow-up data (12 weekends).

Each evaluation procedure included the analysis of previous clinical reports, a family interview, a clinical observation of the child and the application of the battery of instruments, in the presence of, and with the participation of the family and sometimes the children’s teachers and therapists.

Each child underwent a weekly program of twelve sessions, spending 15 min in the water. The interactions were conducted inside a 1150 m^2^ covered artificial lagoon, with the water maintained at 18°C, in a closed section measuring 10x9.5 m, using mostly the shallow area.

The individual animals used for the interaction sessions were selected by the trainer, after observation of their behaviour and motivational states, from a group of common bottlenose dolphins (*Tursiops truncatus*). These animals are maintained in a technically advanced husbandry and care regime, which has received several international awards for training and welfare excellence. They normally participate in a commercial “swim-with-the-dolphins” program, for which regular training sessions are run, based on positive reinforcement methods [30]. The animals’ attention is focused on their trainer’s signals, even when they are in the water interacting with other persons.

In this study, each child entered the water accompanied by a clinical psychologist and a dolphin trainer, who facilitated the contact between the child and the dolphin. Children and adults wore neoprene suits and boots for thermal comfort and protection.

Each child entered the pool by the hand of the clinical psychologist, slowly approaching the dolphin and the trainer. The clinical psychologist only intervened when the children showed fear, anxiety or behaviours that could disrupt the interaction. The trainer played an essential part on promoting the interaction, from simple actions like touching the dolphin to progressively more complex ones such as feeding the dolphin, or swimming with the dolphin (see behaviours listed on Additional file 2: Appendix B). The interaction context was kept as playful and pleasant as possible. If a child showed resistance to a given task, a different task was proposed. If a dolphin showed distress, the trainer released the animal from the interaction pool section and called a different one that displayed more motivation to participate. During this program no aggressive behaviours were exhibited by any dolphin at any time, and only minor episodes of child agitation occurred, with no consequences. Up to six months after the experimental interactions, no viral, bacterial or parasite infections linkable to the program had been detected in either the subjects or the dolphins involved. Concurring with Trone et al. [31], no alteration on the dolphins’ welfare indicators was noted, as the dolphinarium staff repeatedly confirmed. In fact, both the subjects and the dolphins were carefully handled, with priority given to their welfare and security. However, it must be emphasized that this study did not intend to justify the keeping of dolphins in captivity, neither does it support ‘Dolphin-Assisted Therapies’.

All the evaluation and interactions sessions were videotaped and photographed. The interaction videos were later analyzed by two observers, who rated the children’s behavioural complexity according to the ‘Interaction Evaluation Grid’. Five hundred and sixty hours were spent analysing video recordings. It should be stated, at this point, that no member of the research team has the intention of participating in commercial therapeutic programs, nor was any such program in the park’s agenda, even though its board facilitated and even partially funded the research program.

### Statistical data analysis

The effects of the interaction program on the scores of CARS, PEP-R, ATEC, the custom-made ToM protocol and the Interaction Evaluation Grid were evaluated with a Linear Mixed Model [32]. Scores were checked for normality and a compound symmetry covariance structure was assumed based on the lowest Akaike Information Criterion (AIC) and Scharwz’s Bayesian Criterion (BIC) [33]. The baseline and the three follow-up evaluations were taken as repeated measures on the 10 children. The children’s age was inputted in the model as a covariate. No significant effect of age was found; therefore this variable was dropped from subsequent analysis. Data analysis was carried out with SPSS (v. 15, SPSS Inc, Chicago, IL) and statistically significant effects were assumed for *p* <0.05.

## Results

The effects of the interaction program on the ASD children’s development, as evaluated by CARS, PEP-R, ATEC, ToM Task Scale and the Interaction Evaluation Grid, resulted in the data that follows.

**Figure 2:**
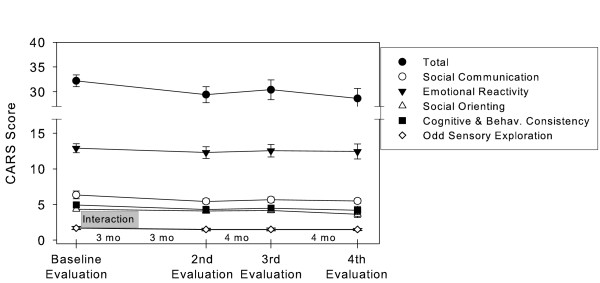
Scores of the total CARS and CARS’ factors. Values are the mean (± SEM) of 10 subjects.

The interaction program showed no significant effects on either the CARS’ total score (F(3,24.407) = 2.15; *p* = 0.120), or on the following factors (after Stella, 1999): Social Communication (F(3,24.504) = 1.73; *p* = 0.187), Emotional Reactivity (F(3,24.484) = 0.257; *p* = 0.856), Social Orienting (F(3,24.351) = 1.861; *p* = 0.163), Cognitive and Behavioural Consistency (F(3,24.4) = 1.483 *p* = 0.244) and Odd Sensory Exploration (F(3,24.348) = 1.022; *p* = 0.400), as presented on Figure 2.

However, the CARS’ single items inferential analysis identified a statistically significant effect on the Non-verbal Communication item (F(3,24.407) = 2.151; *p* = 0.022).

**Figure 3:**
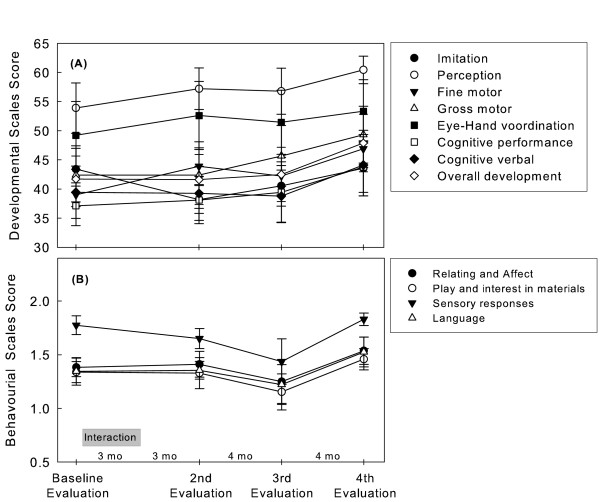
PEP-R Developmental scales (A) and Behavioural Scales (B) scores. Values are the mean (± SEM) of 10 subjects.

Changes in the PEP-R’s Overall development score, Developmental sub-scales and Behavioural sub-scales are presented on Figure 3. Significant results were found on the Overall developmental score (F(3,25.041) = 7.829; *p* = 0.001) and in the Developmental sub-scales Fine motor development (F(3, 25.174) = 4.54; *p* = 0.011), Cognitive performance (F(3, 25.079) = 4.333; *p* = 0.014) and Cognitive verbal (F(3, 25.042) = 5.231; *p* = 0.006). These significant changes are apparent only after the third evaluation. There were no statistically significant changes on Imitation, Perception, Gross motor and Eye-hand coordination, neither on any of the Behavioural sub-scales.

**Figure 4:**
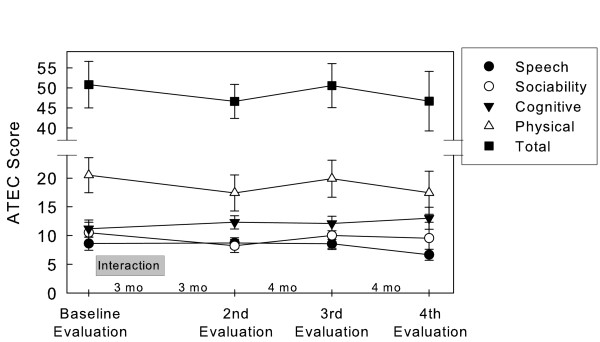
Scores of ATEC. Values are the mean (± SEM) of 10 subjects.

The scores of the ATEC’s four sub-scales, as well as the total score, are presented on Figure 4. No significant changes were observed on the total ATEC score (F(1,25.060) = 0.572; *p* = 0.639), as well as on the Speech (F(1,25.318) = 2.194; *p* = 0.113), Sociability (F(3,24.789) = 1.109; *p* = 0.364), Sensory/Cognitive Awareness (F(3,24.943) = 0.611; *p* = 0.614) and Health/Physical/Behaviour (F(1,25.072) = 1.088; *p* = 0.372) sub-scales.

**Figure 5:**
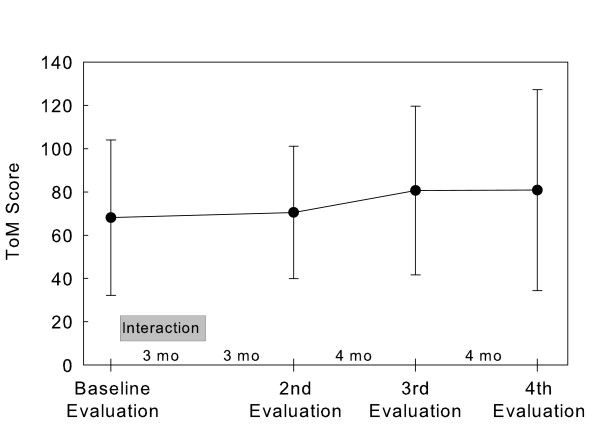
ToM Task Scale scores. Values are the mean (± SEM) of 10 subjects.

The ToM Task Scale score (Figure 5) did not show significant change (F(3, 9.034) = 1.118; *p* = 0.392).

**Figure 6:**
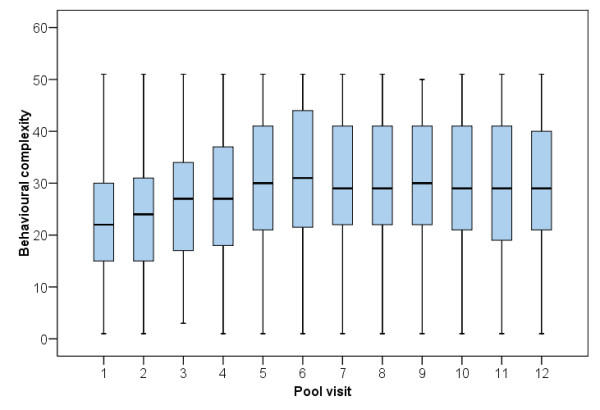
**Box-plots for the children’s behavioural complexity throughout the inside-the-pool interaction sessions.** Top-whisker represents the maximal behavioural complexity for the children (up to 51 behaviours – see Methods); bottom-whisker represents the minimal behavioural complexity. The lower box limit represents the 1^st^ quartile and the top box limit represents the 3^rd^ quartile. The middle line in the box represents the median.

As measured by the ‘Interaction Evaluation Grid’, the evolution of the children’s behavioural complexity inside the pool is illustrated by the Box-plots of Figure 6. The complexity of the behaviours increased significantly (F(11,7346) = 34.014; *p* < 0.001) up to the 6^th^ session, with no significant variation afterwards.

## Discussion

Despite the popularity of dolphin interaction programs with various therapeutic objectives, there is a dearth of clinical effect data using the accepted standardized instruments. In this study, we report data collected during an exploratory interaction program with dolphins and ten children diagnosed with ASD. The program intended to evaluate possible effects, on this sample of children, of twelve once-a-week dolphin interaction sessions. The limited number of children, and sessions (including their low frequency), as well as unavoidable concurrent therapeutic and educational activities by the children may have a confounding effect over the interaction results. Notwithstanding, some results did show significant changes on the clinical condition and development of these children, as summarized below, that warrants further research.

As evaluated by PEP-R, significant changes were observed on the children’s ‘Overall development score’, as well as on their ‘Fine motor development’, ‘Cognitive performance’ and ‘Cognitive verbal development’. However, it must be pointed out that these changes were only apparent 11 months after the dolphin interaction, suggesting either delayed effects or extraneous factors.

On the other hand, the ‘Interaction Evaluation Grid’, which records the children’s behaviour during their interaction with the dolphins, showed a significant evolution in behavioural complexity. However, it is also possible that much of the evolution of the children’s behavioural complexity results from increasing familiarity with the setting or from the trainer’s and the psychologist’s ability to facilitate the emergence of more complex interactions. Perhaps this explains the significant increase in the early sessions, with no significant variation afterwards.

According to CARS, the severity of the clinical picture of autism was not globally affected by the program, although there was a statistically significant change on the ‘Non-verbal Communication’ item; we found this interesting in view of the importance of non-verbal communication to the development of intersubjectivity and, later, of language [21], and certainly worthy of further investigation.

As to ATEC, considered by some researchers as an important evaluation tool, it did not reveal any statistically significant therapeutic changes.

Concerning the ToM Task’s adaptation, it is likely that the children became more at ease with each evaluation session, thus facilitating the slight increase in the scores which, however, were not statistically significant. Of course, as in other cases, the failure to achieve statistical significance may have been due to the reduced power of the statistical tests given the small sample size.

The participant ASD children generally enjoyed the interaction program, and all parents, in follow-up interviews, expressed the impression that it had been useful for their children’s development, and raised subjective hopes for their future. This was shown in their sustained motivation in bringing their children for a full year and a half, and in their expression of desire to continue the program. It must be stated here that families were consistently informed of the exploratory, non-therapeutic nature of this program, in an effort to mitigate the expectation effect. As in related, previous research e.g., [8-10], the dolphin interaction setting was clearly important in the shaping of child acceptance and family interest and involvement. However, the suggesting power generated by such special opportunities demands the greatest care from the researcher, as amply discussed by Marino & Lilienfeld [11,12].

## Conclusions

Despite the small, but statistically significant, improvements observed in some domains of the children’s Fine motor development, Cognitive performance and Verbal development, the program did not affect the overall clinical picture of autism. Therefore, this study does not confirm overall significant developmental progress resulting from a dolphin interaction program. In spite of no clear-cut improvements in ASD children, this type of program remains an enjoyable and unique activity, deserving of further research. While obviously not providing a cure for autism, it may still bear complementary therapeutic potential.

## Competing interests

The authors declare that they have no competing interests.

## Authors’ contributions

ES, LA, AB and AIS collected the data; JM analyzed the data; ES, JM and MES planned the experimental design and analysis. All authors participated in the results’ discussion and writing-up of the manuscript. All authors read and approved the final manuscript.

## Supplementary Material

Additional file 1 Appendix A. File with ToM Tasks measurement instruments. Click here for file

Additional file 2 Appendix B. Measurement instrument for *the Interaction Evaluation Grid (Child-Dolphin-Adults)*. Click here for file
