# Supplementary material for: Effects of a dolphin interaction program on children with autism spectrum disorders – an exploratory research
Source: BMC Res Notes. 2012 Apr 26;5:199. doi: 10.1186/1756-0500-5-199 (PMC3468398; doi:10.1186/1756-0500-5-199)
Supplement: Additional file 1 — Appendix A. File with ToM Tasks measurement instruments. [file 1756-0500-5-199-S1.doc]

**Appendix A: ToM Tasks**

Adapted from Howlin et al., 1999, applied in a Portuguese version.

Child’s name:

Date: / / Observation number:

| **Level 1: Recognizing Facial Expression from Photographs** | | |
| --- | --- | --- |
|  | **Success** | **Failure** |
| Can you point the happy face? |  |  |
| Can you point the sad face? |  |  |
| Can you point the angry face? |  |  |
| Can you point the frightened face? |  |  |

| **Level 2: Recognizing Emotion from Schematic Drawings** | | |
| --- | --- | --- |
|  | **Success** | **Failure** |
| Can you point the happy face? |  |  |
| Can you point the sad face? |  |  |
| Can you point the angry face? |  |  |
| Can you point the frightened face? |  |  |

| **Level 3: Identifying ‘Situation-Based’ Emotions** | | | |
| --- | --- | --- | --- |
| **Picture** | **Serie** | **Success** | **Failure** |
| Alan’s daddy buys him a chocolate ice-cream. | H |  |  |
| Josie and Nigel go to the birthday party. | H |  |  |
| Billy is going round and round on the horse | H |  |  |
| Joanne cannot play on her swing. It’s broken. | S |  |  |
| Lawrence is ill, he cannot go shopping with is mum. | S |  |  |
| It’s time for Adam’s granddad to go home. | S |  |  |
| Lesley is waiting for the fireman to rescue her. | F |  |  |
| The truck is driving fast towards Bobby. | F |  |  |
| The shadow on wall looks like a monster. | F |  |  |
| Bill takes Gavin’s ball. Gavin can’t reach it. | A |  |  |
| Debbie snatches Carol’s teddy and runs way with it. | A |  |  |
| Malcom jumps on Peter’s toy car. It’s broken. | A |  |  |

| *Possible answers:*  Happy (H)  Sad (S)  Frightened (F)  Angry (A) | *Example:*  Situation: Debbie snatches Carol’s teddy and runs way with it.  Question: How does Carol feel? (Angry) |
| --- | --- |

| **Level 4: Identifying ‘Desire-Based’ Emotions** | | | |
| --- | --- | --- | --- |
| **Picture** | **Serie** | **Success** | **Failure** |
| Jean wants to go horse-riding.  Jean’s mum takes her to horse-riding school. | H |  |  |
| Luke wants to sail the boat.  Luke’s daddy says ‘Let’s sail the boat!’ | H |  |  |
| Eric wants to go on the train.  Eric and his daddy are going on the train. | H |  |  |
| Claire wants to see some lambs.  Claire’s mum takes her to see some pigs. | S |  |  |
| Matthew wants a toy train.  Matthew’s brother gives him a toy aeroplane. | S |  |  |
| Toby wants some orange juice.  At bedtime daddy makes him some hot chocolate. | S |  |  |

| *Possible answers:*  Happy (H): Fulfilled Desire  Sad (S): Unfulfilled Desire | *Example:*  Desire: Luke wants to sail the boat.  Outcome: Luke’s daddy says ‘Let’s sail the boat!’  Question: How does Luke feel? (Happy) |
| --- | --- |

| **Level 5: Identifying ‘Belief-Based’ Emotions** | | | |
| --- | --- | --- | --- |
| **Picture** | **Serie** | **Success** | **Failure** |
| Sam’s mum buys him some paints. | | | |
| Sam wants some paints.  He thinks mum has bought him some paints. | H |  |  |
| Sam’s mum gives him the tin of paints | H |  |  |
| Brian’s sister is taking him to the swimming pool | | | |
| Brian wants to go to the swimming pool.  He thinks they are going to the pool. | H |  |  |
| Brian’s sister takes him to the swimming pool | H |  |  |
| Betty’s grandma buys Betty a teddy for her birthday. | | | |
| Betty wants a teddy for her birthday.  Betty thinks grandma has bought a teddy for her birthday. | H |  |  |
| Betty’s grandma gives her the teddy for her birthday. | H |  |  |
| Adrian’s mummy buys him a book about cars. | | | |
| Adrian wants a book about trains.  Adrian thinks mummy has bought a car book. | S |  |  |
| Adrian’s mummy gives him the book about cars. | S |  |  |
| It’s time for Adam’s granddad to go to home. | | | |
| Adam wants his granddad to stay.  Adam thinks his granddad is going home. | S |  |  |
| Adam’s granddad says goodbye and goes home. | S |  |  |
| Tina’s sister buys her a picture of some flowers. | | | |
| Tina wants a kitten picture.  Tina thinks her sister has a flower picture. | S |  |  |
| Tina’s sister gives her the flower picture. | S |  |  |
| Matthew’s brother has a toy aeroplane for him. | | | |
| Matthew wants a toy train.  Matthew thinks his brother has bought a train for him. | H |  |  |
| Matthew’s brother gives him the toy aeroplane. | S |  |  |
| At bedtime daddy makes Toby some hot chocolate. | | | |
| Toby wants some orange juice.  Toby thinks daddy has made him some orange juice. | H |  |  |
| Daddy gives Toby the hot chocolate. | S |  |  |
| Jean’s mummy is taking her horse riding. | | | |
| Jean wants to go dancing. Jean thinks they are going dancing. | H |  |  |
| Jean’s mummy takes her horse riding. | S |  |  |
| At bedtime daddy makes Toby some hot chocolate. | | | |
| Toby wants some hot chocolate.  Toby thinks daddy has made him some orange juice. | S |  |  |
| Daddy gives Toby the hot chocolate. | H |  |  |
| Sam’s mummy has bought him a tin of paints. | | | |
| Sam wants some paints.  Sam thinks mum has bought him a toy car. | S |  |  |
| Sam’s mum gives him the tin of paints | H |  |  |
| Eric and daddy are going on the train. | | | |
| Eric wants to go on the train.  He thinks they are going in the car. | S |  |  |
| Eric and daddy go on the train. | H |  |  |

| *Possible answers:*  Happy/Happy (H/H): True Belief/Fulfilled Desire  Sad/Sad (S/S): True Belief/Unfulfilled Desire  Happy/Sad (H/S): False Belief/Unfulfilled Desire  Sad/Happy (S/H): False Belief/Fulfilled Desire | *Example:*  Situation: At bedtime daddy makes Toby some hot chocolate but he doesn’t know about that.  Desire: Toby wants some hot chocolate.  Belief: Toby thinks daddy has made him some orange juice.  Question: How does Toby feel? (Sad)  Desire: Remember, Toby wants some hot chocolate.  Outcome: Daddy gives Toby the hot chocolate.  Question: How does Toby feel? (Happy) |
| --- | --- |
