# Supplementary material for: Effects of a dolphin interaction program on children with autism spectrum disorders – an exploratory research
Source: BMC Res Notes. 2012 Apr 26;5:199. doi: 10.1186/1756-0500-5-199 (PMC3468398; doi:10.1186/1756-0500-5-199)
Supplement: Additional file 2 — Appendix B. Measurement instrument for the Interaction Evaluation Grid (Child-Dolphin-Adults). [file 1756-0500-5-199-S2.docx]

**Appendix B**

**Interaction Evaluation Grid (Child-Dolphin-Adults)**

| 1 | Inadequate interaction with adults |
| --- | --- |
| 2 | Agressive behavior towards the dolphin |
| 3 | Refuses any interaction |
| 4 | Avoids the dolphin |
| 5 | Shows no interest in the dolphin |
| 6 | Expresses displeasure |
| 7 | Refuses to take the dolphin's reinforcement fish |
| 8 | Displeasure in the contact with water |
| 9 | Unbalance/Falls down |
| 10 | Plays outside the lagoon |
| 11 | "Tastes" water |
| 12 | Grabs the adult |
| 13 | Jumps in the water |
| 14 | Pleasure in the contact with water |
| 15 | Swims or dives |
| 16 | Interacts with the dolphin only with help of the adult |
| 17 | Picks up the fish and throws it away |
| 18 | Pays attention to the dolphin's sounds |
| 19 | Shows pleasure in the interaction |
| 20 | Looks towards the adults who are outside the lagoon |
| 21 | Looks to the adults inside the lagoon |
| 22 | Looks at the dolphin |
| 23 | Salutes the dolphin trainer |
| 24 | Salutes the dolphin |
| 25 | Passes through the loop |
| 26 | Plays with the board |
| 27 | Throws the fish near the dolphin's mouth |
| 28 | Touches the dolphin |
| 29 | Caresses the dolphin |
| 30 | Holds the dolphin |
| 31 | Kisses the dolphin |
| 32 | Plays "hide and seek" with the dolphin |
| 33 | Plays "splashing water war" with the dolphin |
| 34 | Promenades with the dolphin |
| 35 | Plays “vacuum cleaner”, holding the caudal fin |
| 36 | Hugs the dolphin |
| 37 | Imitates the adult |
| 38 | Imitates the dolphin |
| 39 | Interacts through objects |
| 40 | Lifts the dolphin with the trainer |
| 41 | Interacts with the adults |
| 42 | Turns the dolphin around with the belly up |
| 43 | "Listens to the heart of the dolphin" |
| 44 | Puts the fish in the dolphin's mouth |
| 45 | Rides in the dolphin's dorsal fin |
| 46 | "Dances" with the dolphin |
| 47 | Accepts dolphin's handpush |
| 48 | Accepts dolphin's footpush |
| 49 | Rides on the dolphin's belly holding its pectoral fins |
| 50 | Gives gestural commands to the dolphin |
| 51 | Complex interaction with the dolphin |
